# How virtual urban green spaces influence stress and risk-taking

**DOI:** 10.3389/fpsyg.2026.1710257

**Published:** 2026-04-01

**Authors:** Sanaz Dorri Sedeh, Vladimir Kosonogov, Nadezhda Kerimova, Kimberly L. Meidenbauer, Vasily Klucharev

**Affiliations:** 1 International Laboratory of Social Neurobiology, Institute of Cognitive Neuroscience, National Research University Higher School of Economics, Moscow, Russia; 2 Department of Psychology, Washington State University, Pullman, WA, United States; 3 Affective Psychophysiology Laboratory, Institute of Health Psychology, National Research University Higher School of Economics, Saint Petersburg, Russia; 4 Department of Landscape Architecture, Saint Petersburg State University of Architecture and Civil Engineering, Saint Petersburg, Russia

**Keywords:** greenery, heart rate, risk-taking, skin conductance response, stress recovery, urban environment, 360° video, Neurourbanism

## Abstract

**Introduction:**

As cities continue to grow, access to natural environments is becoming more limited, contributing to increased stress levels in urban populations. Panoramic 360° videos provide a creative and scalable means of simulating natural environments, potentially reducing stress in city residents under controlled settings. Here, we examined whether short immersive experiences in different urban environments support stress recovery and influence risk-taking behavior.

**Methods:**

Sixty-six participants were subjected to stress inductions using the threat-of-shock paradigm before viewing 360° monoscopic videos of urban walks through a park or a highway, or a minimal-stimulation control video. While participants watched videos using VR headsets, we recorded their heart rate and skin conductance responses. We also collected self-reported stress ratings and assessed risk-taking behavior using the Balloon Analogue Risk Task.

**Results:**

The results showed that self-reported stress was significantly lower following the urban park video compared to both the highway and control conditions. Physiologically, heart rate and skin conductance responses were significantly lower during the park conditions compared to the highway conditions, but did not differ significantly between the park and control conditions. No effects of the 360° video environments were observed on heart rate variability or risk-taking behavior.

**Discussion:**

These findings suggest that brief exposure to virtual urban green spaces supports stress recovery following acute stress, particularly relative to highly arousing urban environments.

## 1 Introduction

Over 50 % of the global population currently resides in urban areas, and this proportion is projected to rise to 70 % by 2050 (Angel et al., 2011; Seto et al., 2012). People who live in urban areas often experience more stress in daily life than residents of rural areas (Hedblom et al., 2019). Furthermore, urban factors such as traffic congestion, and elevated levels of air and noise pollution contribute to the development of various disorders (Groenewegen et al., 2006; Haluza et al., 2014; James et al., 2015). Unlike urban environments, exposure to green spaces has been robustly linked to improvements in mood, relaxation, and overall well-being (Hartig and Kahn, 2016; Kaplan, 1995, 2001; Tyrväinen et al., 2014; Ulrich, 1983; Ulrich et al., 1991). An influential psycho-evolutionary theory suggests that built environments lead to stronger physiological and cognitive depletion than natural environments (Ulrich, 1983; Ulrich et al., 1991). In line with this idea, many studies have suggested that natural environments reliably increase the power of alpha oscillations that are sensitive to the level of relaxation (Chang et al., 2008; Grassini et al., 2019; Roe et al., 2013; Ulrich, 1981) and favor stress reduction and attention restoration (Aspinall et al., 2015; Grassini et al., 2019; Lin et al., 2020). Furthermore, urban green spaces increase brain-to-brain synchrony in the delta band, which reflects the most evolutionary old and phylogenetically preserved cortical activity involved in basic motivation and reward (Kerimova et al., 2025). The current study builds on this literature by examining whether exposure to green urban environments facilitates greater stress recovery compared to build environments. Overall, green spaces create essential cognitive, psychological, and emotional benefits (Lee et al., 2009; Hedblom et al., 2019). There is growing consensus across the natural, social, and health sciences on the positive impacts of urban greenery on stress reduction and mental health (Bratman et al., 2019). Furthermore, a summary of previous studies by the World Health Organization clearly indicates that green spaces facilitate urban health and well-being (WHO, 2016). Green urban spaces include trees along the streets, public parks, national parks, local parks, private gardens, forests, and even playgrounds. Lindal and Hartig (2015) concluded that green urban spaces improve air quality, reduce stress, and stimulate physical activity and social participation. Furthermore, Lee et al. (2011) demonstrated a correlation between forest exposure and immune system improvement, such as the production of anti-cancer proteins. Large green spaces, such as open spaces with natural features and vegetation, such as parks, provide effective conditions for stress restoration (Van den Berg et al., 2015a). Several studies have demonstrated the positive impact of urban green spaces on mitigating stress. For instance, individuals residing in low-income urban neighborhoods and dealing with chronic stress reported experiencing stress reduction, specifically when they visited green urban areas that lowered levels of cortisol, heart rate (HR), and blood pressure compared to build urban spaces (for a review, see Maas et al., 2009). Exposure to green spaces has also been associated with stronger activation of the parasympathetic nervous system and inhibition of the sympathetic nervous system (Mygind et al., 2021; Tsunetsugu et al., 2013; Hedblom et al., 2019). Recent research indicates that exposure to green areas can affect brain activity in the subgenual prefrontal cortex, which is involved in emotional regulation and reward mechanisms, leading to a reduction in depression and stress levels (Bratman et al., 2019). Taken together, previous research indicates that green spaces help to reduce stress and generally enhance psychological recovery.

Although the stress-reduction effects of natural environments are well-known, a growing body of research indicates that nature exposure may also influence decision-making processes, particularly risk-taking behavior (e.g., van der Wal et al., 2013; Berry et al., 2014). In fact, the stress reduction in natural environments can mediate the effects of nature on risk-taking, because the direct link between stress and risk-taking has been robustly demonstrated. For example, the extensive meta-analysis that investigated decision making after a stress induction versus a control condition revealed that stress conditions reliably lead to more reward-seeking decisions and more risk-taking than nonstress conditions (Starcke and Brand, 2016). Interestingly, several studies have found evidence linking natural images to reduced impulsivity (van der Wal et al., 2013; Berry et al., 2014; Berry et al., 2015; Repke et al., 2018) in tasks that have been consistently associated with impulse control including risk attitudes and risk-taking (e.g., Mishra et al., 2017; Mishra and Lalumière, 2017). Furthermore, results of a recent questionnaire survey suggested that nature connectedness can effectively moderate Internet Gaming Disorder via the mediating effect of intolerance of uncertainty (Yuan et al., 2024). In addition, a growing number of studies suggest that residential greenspace is associated with a lower prevalence of health risk behaviors (e.g., Martin et al., 2025). Overall, prior studies allow one to hypothesize that impulsive risk-taking mediates the nature–health relationship, to some extent (Repke et al., 2018).

Risk-taking is a fundamental aspect of human behavior that encompasses decisions made under uncertainty, in which potential rewards must often be weighed against possible losses. From an evolutionary perspective, adaptive risk assessment is crucial for survival, influencing foraging decisions, territory selection, and resource allocation (Houston et al., 2014). In modern urban contexts, risk-taking manifests in various domains, including financial decisions, health behaviors, and social choices (Lejuez et al., 2002). Exposure to natural environments has been associated with reduced impulsivity and delay discounting, important components of risk-taking behavior (Berry et al., 2014; Panno et al., 2021; Van der Wal et al., 2013) by reducing impulsivity and delay discounting the tendency to prefer smaller, immediate rewards over larger, delayed ones. Overall, natural settings appear to promote more deliberate, conservative decision-making compared to urban environments. Similarly, time spent in natural environments has been linked to decreased health-risk behaviors, including substance misuse and aggression (Kuo and Sullivan, 2001; Van der Wal et al., 2013). The mechanism underlying these effects of natural environments might involve not only the stress reduction, but also restoration of executive function and self-regulation capacities. By contrast, urban environments impose cognitive demands that can deplete regulatory resources, leading to more impulsive decision-making (Berman et al., 2008; Berry et al., 2020). This behavioral shift likely stems from the restoration of self-regulatory capacities. Acute stress and urban environments impose cognitive demands that deplete executive functions required for impulse control. For example, according to Attention Restoration Theory (ART), natural environments replenish these resources by providing ‘soft fascination’ and relief from mental fatigue (Kaplan, 1995, 2001). Consequently, the physiological recovery promoted by green space should facilitate a shift from impulsive reward-seeking toward more deliberate risk assessment.

In the current study, we used the Balloon Analogue Risk Task (BART) as a conventional behavioral measure of risk-taking to further probe the behavioral effects of urban greenery. In the BART, participants inflated virtual balloons to earn rewards, facing an increasing risk of explosion and loss of all trial earnings with each pump. This task models real-world risky decision-making by requiring participants to balance the potential for increasing rewards against the escalating risk of loss. Importantly, riskiness on BART has been associated with sensation seeking and impulsivity, as well as with self-reported addictive, health, and safety risks (Lejuez et al., 2002). Therefore, based on the previous literature, we hypothesized that exposure to green urban environments lowers risk-taking in BART compared to exposure to built environments. Most previous studies that were conducted in laboratory settings to examine the impact of greenery on stress have primarily relied on the use of pictures or images (Bratman et al., 2019; Brown et al., 2013; Herzog et al., 2003; Ulrich et al., 1991; Van den Berg et al., 2015b). Advancements in technology allow us to utilize more ecologically valid stimuli, such as 360° videos, to study the effects of greenery on stress (e.g., Newman et al., 2022). Recent research has shown that immersive exposure to urban green infrastructure, such as parks and greenways, can reduce stress and improve mental health outcomes, supporting the use of immersive urban nature interventions (Jiang et al., 2024).

Real nature alleviates anxiety, stress, and depression (Liu et al., 2023; Paredes-Céspedes et al., 2024; Pun et al., 2018) and offers health benefits across the entire life span (Bray et al., 2022; Browning et al., 2023; Gritzka et al., 2020; Pun et al., 2018). However, rapid urbanization has limited nature contacts dramatically (Kellert, 2017; Maddock and Johnson, 2024; United Nations, Department of Economic and Social Affairs, Population Division, 2015). Nevertheless, technological innovations may help to overcome these limitations (Choi et al., 2022), and a growing number of studies demonstrate the positive impact of immersive nature on individuals with depression, anxiety, and dementia (Lee et al., 2024; Li et al., 2021; Reynolds et al., 2018). Importantly, a recent meta-analysis indicated that exposure to Virtual natural environments effectively reduces anxiety, stress, and depression in healthy adults (Chen et al., 2025). These findings strongly suggest that immersive Virtual natural environments have a positive effect on mental health and can be useful when direct access to natural environments is limited.

Unlike 360° photographs, which offer only a fixed, limited perspective, 360° movies allow users to move through the urban environment, creating a greater sense of presence and realism. Importantly, immersive environments trigger more lifelike physiological responses than traditional photos (Kisker et al., 2021). Therefore, the current study further investigated the effect of 360° monoscopic videos of green urban environments on stress recovery. Important advantages of panoramic 360° videos also include temporal realism – the unfolding of environmental events over time mirrors real-world experiences (Chirico and Gaggioli, 2019) and the sensation of moving through rather than merely observing an environment, promoting deeper psychological and physiological engagement (Song et al., 2022; Freeman et al., 2017).

Several studies have demonstrated the effectiveness of full 360-degree images or videos of nature in studies of green environments. For example, a recent study showed that a VR headset and virtual nature room had a better impact on psychological stress recovery than a TV delivery of virtual nature (Kari et al., 2024). Another study employed a between-group design and minor electrical shocks to demonstrate that exposure to virtual multisensory (visual and auditory olfactory stimuli) 360° images of parks and forests led to a reduction in skin conductance levels, indicating a decrease in stress responses (Hedblom et al., 2019). A study that utilized stress-inducing (memory and arithmetic) tasks, along with 360° images of offices, showed that exposure to biophilic indoor environments helped people recover from stress and anxiety and that the restorative effects differed among distinct types of biophilic elements (Yin et al., 2020). Moreover, the nature-based VR environment led to reductions in self-reported and physiological measures of stress, including skin conductance and HR (Valtchanov and Ellard, 2010). Some previous studies have suggested that both panoramic photos and real environments can provide similar healing benefits (Song et al., 2022). Importantly, immersive images of nature can also encourage individuals to enjoy nature outside (Yu et al., 2018). Additionally, high-realism videos can elicit more positive affective responses and perceptions than watching a traditional video, simulating a real-world experience and evoking higher feelings of serenity, enjoyment, presence, and immersion (Newman et al., 2022). Here, we aimed to further explore the effects of 360°-videos of urban environments on both stress reduction and risk-taking behavior. Most previous studies of stress reduction have focused on forests, lawns, and meadows (Li et al., 2021; Valtchanov and Ellard, 2010; Chan et al., 2023; Yin et al., 2020), using 2D and 360° photos (Brown et al., 2013; Van den Berg et al., 2015b) with a limited number of control conditions. Furthermore, these studies often used between-subject designs (Hedblom et al., 2019; Li et al., 2021; Liu et al., 2022; Van den Berg et al., 2015b), artificial images (Newman et al., 2022), or social stressors (Annerstedt et al., 2013; Van den Berg et al., 2015b). In the current study, we used a within-subject design to examine the stress reduction effects of monocular 360° videos of real urban environments, including a representative green city park and a highway that lacked urban greenery. Our participants were exposed to dynamic monoscopic 360° videos featuring continuous motion that simulated a first-person walking experience through urban environments. We also included an additional baseline (control) condition to control for the complexity of the video stimulation. To study the effects of 360° videos on stress reduction, we used a standard threat-of-shock (ToS) paradigm to provoke a stressful anxiety state and allow multiple repetitions of the stress induction (Robinson et al., 2019; Sarigiannidis et al., 2020; Schmitz and Grillon, 2012). Each stress induction session was followed by a 5-min dynamic 360° videos of the green environment, built environment, or control video with no visual stimulation. During the videos, we recorded skin conductance response (SCR), HR, Decision-making under uncertainty behavior (BART), and self-reported levels of stress.

To sum up, we hypothesize (Hypothesis H1) that exposure to a 360° video of an urban park would result in greater stress recovery measured by skin conductance response (SCR), heart rate (HR), heart rate variability (HRV), and self-reported stress than exposure to a 360° video of a highway environment. We also hypothesize (Hypothesis H2) that exposure to a 360° video of a green urban park would lead to lower risk-taking in the BART task compared to exposure to a 360° video of a highway.

## 2 Materials and methods

### 2.1 Participants

Sixty-six participants (ages 19–42; M = 23.76, SD = 6.38; 37 females, 29 males) were recruited through public advertisements on social media platforms. The sample included 47 undergraduate students and 19 graduate students, including three doctoral (PhD) candidates. All participants reported normal or corrected-to-normal vision and no history of psychiatric, neurological, cardiovascular, or cognitive disorders. Each participant provided written informed consent prior to participation. The study protocol was approved by the local university ethics committee (No.99, December 13, 2022). Upon completion of the study, the participants received a compensation of $10.00 USD plus performance bonuses based on points earned during the BART task. The sample size was determined based on effect sizes reported in similar VR stress recovery studies (e.g., Browning et al., 2020), and a target sample of 60 was selected to ensure adequate power (80%) to detect medium effects (*f* = 0.25) in a repeated-measures ANOVA with three conditions. No formal *a priori* power calculation was conducted.

### 2.2 Study design

We employed a within-subject design to control intersubject variability (Van den Berg et al., 2015b). Each participant underwent three experimental blocks, along with a baseline block, comprising multiple trials. The blocks included BARTs (Lejuez et al., 2002), stress induction using the ToS paradigm (Hedblom et al., 2019; Schmitz and Grillon, 2012), and the video of a 360°-video urban environment. Physiological indices of stress, including photoplethysmography (PPG) for measuring HR and SCR, were recorded throughout the experiment. We used PPG as an optical measurement to detect the blood pressure pulse. By measuring the light absorption of arterial blood, PPG provides a reliable proxy for autonomic nervous system activity through the analysis of pulse rate intervals.

Overall, such a within-subject design eliminates individual differences and prevents these extraneous variables from confounding the results, which can be particularly important in studies using stress induction. To mitigate potential practice or learning effects, we employed a Latin square counterbalancing procedure for the 360°-video condition. These clarifications have been added to the Experimental Design subsection. With individual variability removed as a factor, we could more confidently attribute observed changes in the dependent variable to the manipulation of the independent variable.

#### 2.2.1 Procedure

At the beginning of the study, after reading the instructions, a pair of silver chloride electrodes was attached to each participant’s hands. The participants were informed that they would receive mild electrical shocks on the index and middle fingers of their dominant hand. Next, the appropriate level of electrical stimulation was determined (see details below). Physiological sensors were then attached to the participants’ hands, including PPG sensors on the middle finger and SCR sensors on the index and ring fingers.

Following this setup, the participants completed a baseline block, which included the ToS induction session, followed by self-report measures, and BARTs to establish baseline risk-taking behavior. The participants then completed three experimental blocks, like the baseline block, featuring 360° monoscopic videos of urban walks through a park or highway, or a control video in a VR setting. The order of these experimental blocks was randomized across participants. The order of BART trials was randomized across blocks to further reduce learning effects. We used a Latin square design to counterbalance the sequence of video conditions (park (P), highway (H), control (C)), ensuring that each condition appeared equally often in each ordinal position (first, second, third). Specifically, the participants were manually assigned to one of six predetermined sequences: PHC, PCH, HPC, HCP, CHP, and CPH. This approach is minimized with potential order effects and distributional biases. Additionally, the participants took a 30-s inter-block break, during which the researcher assisted them in putting on the VR headset, ensuring comfort and readiness for the immersive experience. After the video task, another 30-s break allowed the researcher to help the participants remove the headset, facilitating a smooth transition back to the experimental environment. Each experimental block followed the same structure across each block.

Importantly, participants had full rotational freedom, allowing them to freely turn their heads and visually explore the environment, while their physical position remained fixed and no translational movement was permitted.

**Figure 1:**
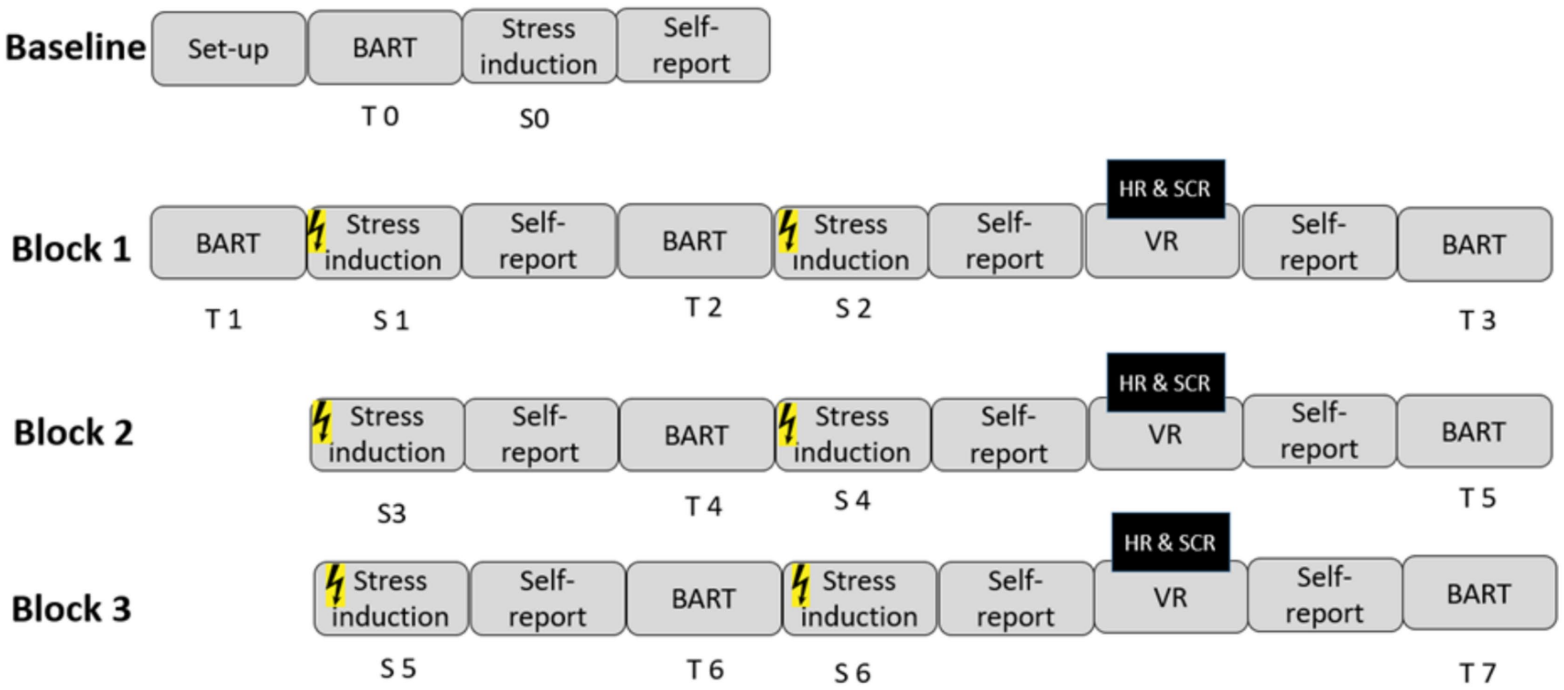
Study design. The baseline block included an initial Balloon Analogue Risk Task (BART), followed by threat-of-stress (ToS) induction, a self-report measure, and a second BART. The subsequent three experimental blocks. The order of BART trials was randomized across blocks to further reduce learning effects. In each experimental block, the participants used a VR headset and viewed a 5 min 360° video of either a park, a highway, or a control video with no visual stimulation. The presentation order of the 360° videos was randomized across the participants. Black squares indicate the time windows during which physiological responses (heart rate [HR] and skin conductance response [SCR]) were analyzed.

Figure 1 illustrates the study design. In Block 1, the participants completed a pre-stress BART (T1), a 5-min ToS induction session (S1), and a self-report stress survey. Next, a second BART was performed (T2), followed by a second 5-min ToS induction session (S2) and another self- report stress survey. The participants were then exposed to a random environment while their heart activity (HR) and skin conductance (SCR) were recorded. After 360° video exposure, the participants completed a final self-reported stress survey. In Block 2, the participants completed a pre-stress BART (T3), then a 5-min ToS induction session (S3), followed by a self-report stress survey, the second BART (T4), the second 5-min ToS induction session (S4), and another self-report stress survey before exposure to the 360° videos (while HR and SCR were recorded). Block 3 mimicked this same approach with a pre-stress BART (T5), stress induction (S5), self-reported stress, the second BART (T6), stress induction (S6), stress self-report, and 360° video exposure. Finally, the participants completed a post-VR BART (T7) questionnaire to measure any lasting effects on risk-taking behavior. Throughout the experiment, the participants were instructed to watch the videos attentively. This design allowed for the inclusion of two ToS induction sessions in each block and the collection of accurate baseline behavioral measurements across all blocks.

### 2.3 Visual stimuli – urban and control environments

In the current study, we used three (5-min) 360° monoscopic videos representing different urban environments: a park, a busy highway, and a control condition. All videos were recorded on the same day using a Ricoh Theta camera under similar lighting and weather conditions. All footage was edited using the VSDC Video Editor to ensure a consistent video length (5 min) and pacing. Importantly, participants did not watch the video on a standard screen, the 360-degree video was shown through a VR headset, which allowed free head movement and provided a stronger immersive viewing experience.

**Figure 2:**
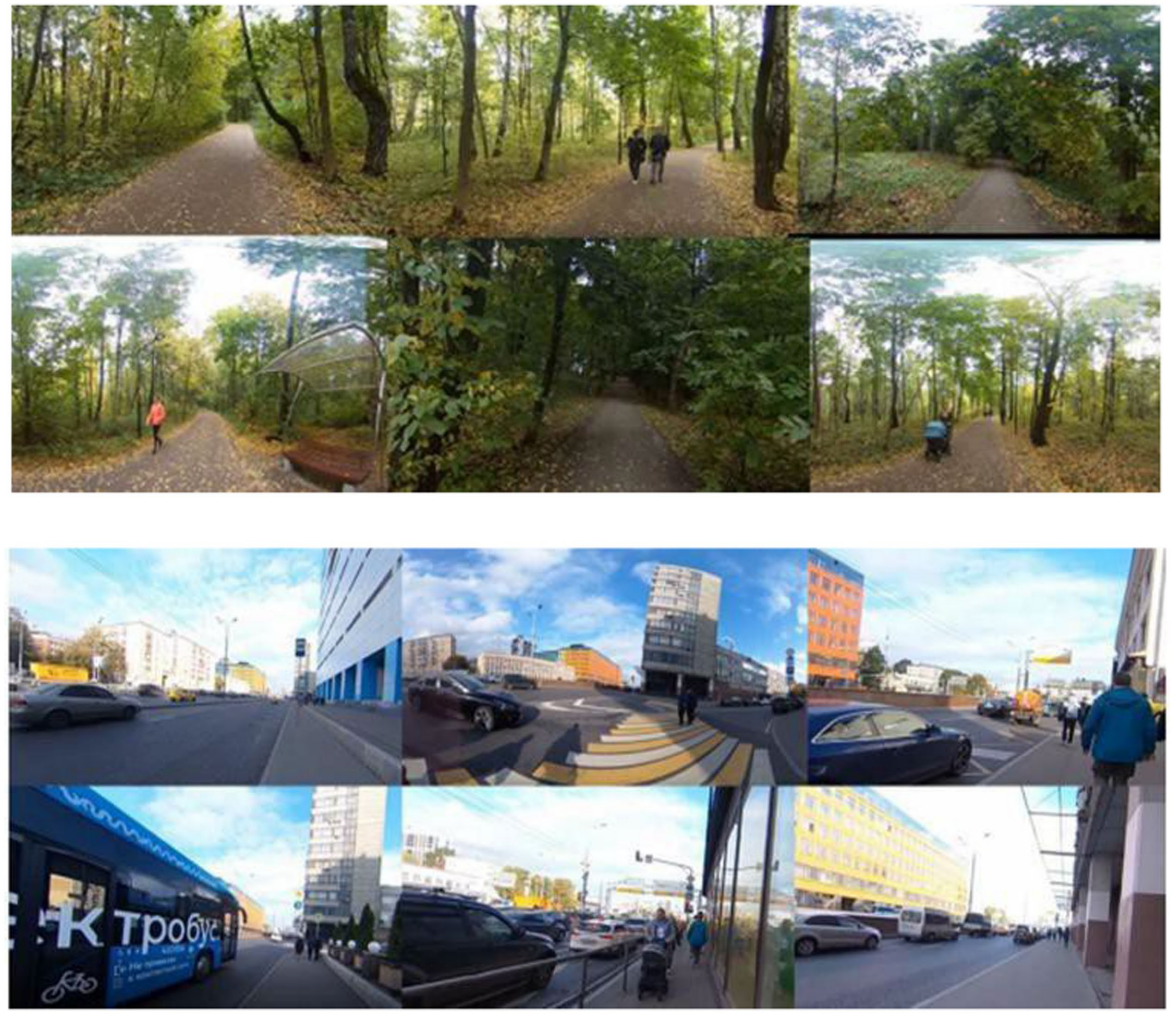
Representative frames from the 360° (5-min) video of the park (top) and the highway (bottom).

The park video was recorded in a centrally located urban green space surrounded by residential and commercial buildings. The footage includes benches, paved trails, grassy areas, occasional passersby, and glimpses of nearby infrastructure, capturing the 5-min experience of walking through a real, accessible city park. By contrast, the highway video depicts a roadside walk-through in a dense urban zone with heavy traffic, concrete infrastructure, and minimal greenery (Figure 2).

The control condition consisted of a black screen with a white fixation cross presented in a 360° video format. Although visually minimal, this condition was delivered via the same VR headset to ensure that the immersive setup (including the spatial orientation and headset display properties) was held constantly across the conditions. The black-screen condition served as a minimal-stimulation reference condition delivered through the same VR headset, allowing us to standardize display properties and isolate the effects of the park and highway content from headset-related factors. This condition was intended to provide a neutral, low-information comparison and should not be interpreted as an ecologically matched urban control stimulus.

All videos were viewed using an HTC Vive Pro head-mounted display, which featured two 3.6- inch screens with a resolution of 1,080 × 1,200 pixels per eye (2,160 × 1,200 combined), a 90 Hz refresh rate, and a 110-degree field of view. To minimize distractions, the participants were placed in an immersive panoramic video-environment with limited external noise or visual input. The videos were full-motion panoramic recordings simulating real-time movement through the environment rather than static images, allowing the participants to explore by turning their heads. The stimuli provided a naturalistic and ecologically valid experience, although they were non-interactive (Mattila et al., 2020).

### 2.4 The balloon analogue risk task

BART is frequently used in research to assess an individual’s risk-taking tendencies (Lejuez et al., 2002). Previous studies have shown that BART results correlate with various risk-taking behaviors, including alcohol consumption, cigarette and drug use, gambling, unsafe sexual practices, and sensation-seeking activities (Lejuez et al., 2002). In the present study, the computerized version of BART was programmed using PsychoPy software (version 2022.2.2; Peirce, 2009). Risk-taking behavior was measured as the average number of balloon inflation pumps per trial, which was directly related to the likelihood of “popping” the balloon.

During each trial, the computer screen displayed a balloon, and each click of the “pump” button inflated the balloon. The number of monetary units (MUs) earned per pump varied across trials: eight balloons had a low value (0.5 cent per pump), eight balloons had a medium value (1.0 cent per pump), and eight balloons had a high value (5.0 cents per pump). The number of earned MUs increased with each pump until either (a) the balloon “popped,” causing the participants to lose their earnings for that trial, or (b) the participants chose to stop inflating the balloon and “cash out,” collecting their accumulated MUs. The MUs ranged from 0 to 120, with 1 MU equaling 0.04 USD (according to the Big Mac index) (Lejuez et al., 2002). The participants were informed that the balloon could explode at any time during inflation and that a randomly selected trial would yield an additional monetary reward. They were not given detailed information regarding the probability of the balloon exploding. In addition to a fixed base payment for participation (about $10.00), participants earned real monetary rewards based on their performance in the BART, such that their risk-taking decisions involved actual financial incentives. of $10.00. Every participant ended up with additional earnings from about US $5 to $10.

To study the effect of the urban environment condition on risk-taking, we subtracted the results of the BART trials following each panoramic urban walk (e.g., T3 in the Block 1) from the results of the BART trials preceding each panoramic urban walk (e.g., T2 in the Block 1) (i.e., T2 – T3; Figure 1). To assess the effect of stress on risk-taking, we subtracted the results of the second block of the BART trials following the ToS induction (e.g., T4 in the Block 2) from the results of the first block of BART trials preceding the ToS induction (e.g., T3 in the Block 2) (i.e., T3 – T4; for details, see Figure 1). The trial order within BART was randomized to minimize learning effects across repeated blocks. This randomization helped ensure that the observed differences were due to experimental manipulation rather than task familiarity.

### 2.5 Stress induction protocol

Stress can be reliably elicited in healthy individuals using the ToS paradigm (Robinson et al., 2013; Sarigiannidis et al., 2020; Schmitz and Grillon, 2012). The ToS has been validated across numerous behavioral and clinical studies as an effective method for inducing a sustained state of anxiety (Robinson et al., 2013). During ToS sessions, the participants anticipate intermittent and unpredictable aversive electrical stimulation, which reliably evokes self-reported distress, physiological arousal, and neurobiological markers of anxiety (Robinson et al., 2019; Schmitz and Grillon, 2012).In the present study, two adhesive surface electrodes were attached to the participants’ dominant hand, and electrical pulses were administered via a D185 Digitimer MultiPulse Transcranial Cortical Stimulator. The procedure followed standard protocols from prior research (Hedblom et al., 2019; Sarigiannidis et al., 2020). Each ToS session included two blocks separated by a one- minute break. During each block, a red square (stimulus duration = 8 s) appeared on the screen (23-inch LG monitor) six times, with interstimulus intervals ranging from 8 to 20 s. An electrical shock followed the stimulus in 50% of the trials. The participants received three shocks per block, resulting in 18 shocks across the entire experiment.

#### 2.5.1 Electrical stimulation calibration

Prior to the main study, we determined each participant’s individual threshold for aversive electrical stimulation during a 15-min calibration session. A series of electrical pulses (duration = 5 ms), starting at 20 volts (approximately 1 mA), were administered with gradually increasing intensity. After each pulse, the participants rated its intensity and the anticipated intensity of the next pulse using a 10-point Likert scale (1 = barely felt, 10 = very unpleasant/uncomfortable). The procedure continued until the participant rated a pulse as very unpleasant/uncomfortable (i.e., a score of 10), at which point the calibration was terminated. The voltage level at this threshold was then used as the aversive stimulus in the ToS paradigm during the main study.

### 2.6 Psychological and physiological measures of stress

After each ToS and Video session, the participants evaluated their current emotional state using a 9- point Likert scale ranging from 1 (high calmness) to 9 (high stress). Emotional state was assessed using this single-item self-report scale, which has been used in prior research as a face-valid, rapid assessment of state stress (e.g., Kirschbaum et al., 1993).

Skin conductance response (SCR) and cardiac activity were recorded at a sampling frequency of 500 Hz using the NVX36 system (Medical Computer Systems) and processed with NeuroKit (Makowski et al., 2021), a Python-based physiological signal analysis library. The tonic component of skin conductance was removed, after which peaks were identified and their amplitudes summed over each 5-min epoch for each Video condition. For each 5-min immersive video, we calculated the mean HR, HRV, standard deviation of NN intervals (SDNN), low- frequency to high-frequency ratio (LF/HF), and Poincaré plot–based HRV parameters: SD1, SD2, and the SD1/SD2 ratio.

The participants’ heart rates were collected using photoplethysmography (PPG) sensors attached to the middle finger of the non-dominant hand. PPG was selected for its non-invasive nature and compatibility with VR equipment. Whereas electrocardiography (ECG) offers greater precision, PPG has been validated as a reliable alternative for capturing HR and HRV in similar contexts (van Gent et al., 2019). Physiological sensors were attached to the participants at the beginning of the study: PPG was recorded using the middle finger, and SCR sensors were attached to the index and ring fingers of the dominant hand. A 30-s break was provided between each block to reduce potential carryover effects. After each ToS and Video session, the participants evaluated their current emotional state using a 9-point Likert scale ranging from 1 (high calmness) to 9 (high stress). Skin conductance response (SCR) and cardiac activity were recorded at a sampling frequency of 500 Hz using the NVX36 system (Medical Computer Systems) and processed with NeuroKit (Makowski et al., 2021), a Python-based physiological signal analysis library. The tonic component of skin conductance was removed, after which peaks were identified and their amplitudes summed over each 5-min epoch for each Video condition. For each 5-min 360° video, we calculated the mean HR, HRV, SDNN, LF/HF, and Poincaré plot–based HRV parameters: SD1, SD2, and the SD1/SD2 ratio. Physiological sensors were attached to the participants at the beginning of the study: PPG was recorded using the middle finger, and SCR sensors were attached to the index and ring fingers of the non-dominant hand.

### 2.7 Statistical analysis

We used one-way analysis of variance (ANOVA) with repeated measures to compare self-reported stress, skin conductance (the sum of peak amplitudes), HR, and HRV in three experimental conditions (factor *Video environment*: (1) video walk through the park, (2) video walk through the highway, or (3) video walk through the control environment). *Post hoc* pairwise comparisons were performed with Tukey correction (Hsu, 1996). Statistical analysis was performed using *Jamovi 2.3.24* (Richardson and Machan, 2021).

To study the effect of urban environment conditions on risk-taking, we subtracted the results of the BART trials that were collected immediately after each video urban walk from the results of the BART trials that preceded each video urban walk (i.e., T2-T3, T4-T5, and T6-T7; for details, see Figure 1). To study the effect of ToS induction on risk-taking, we subtracted the results of the second BART in each block that were collected after the ToS induction from the results of the first BART in each block that preceded the ToS induction (i.e., T1-T2, T3-T4, and T5-T6, for details, see Figure 1).

## 3 Results

### 3.1 Self-reported stress

**Figure 3:**
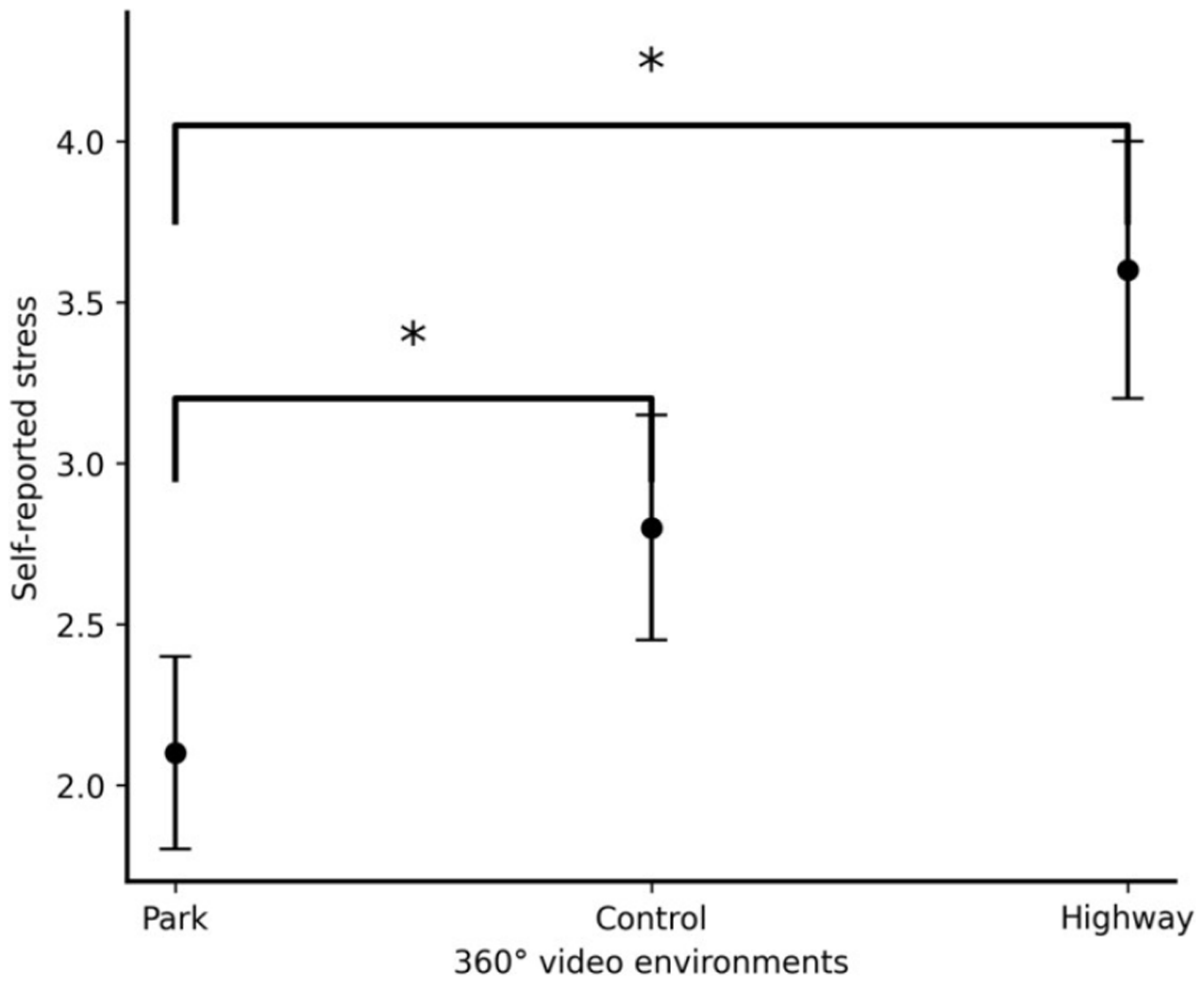
The graph illustrates the estimated marginal means of self-reported stress levels (referred to as “Self-reported Stress”) reported after exposure to 360° monoscopic videos of distinct environments: park, control, and highway. An asterisk (*) denotes statistically significant differences between the environments. Error bars represent standard error of the mean (SEM).

A one-way repeated-measures ANOVA revealed a significant effect of the Video environment on self-reported stress: *F* (2, 130) = 28.10, *p* < 0.001, η^2^_p_ = 0.302 (see Figure 3). *Post hoc* tests indicated that self-reported stress was significantly lower after the video walk through the park compared to the control stimulus: *t* (65) = −4.07, *p* < 0.001, *d* = 0.50, and the video walk through the highway, *t* (65) = −8.22, *p* < 0.001, *d* = 1.01.

### 3.2 Physiological measures

#### 3.2.1 Heart rate and heart rate variability

**Figure 4:**
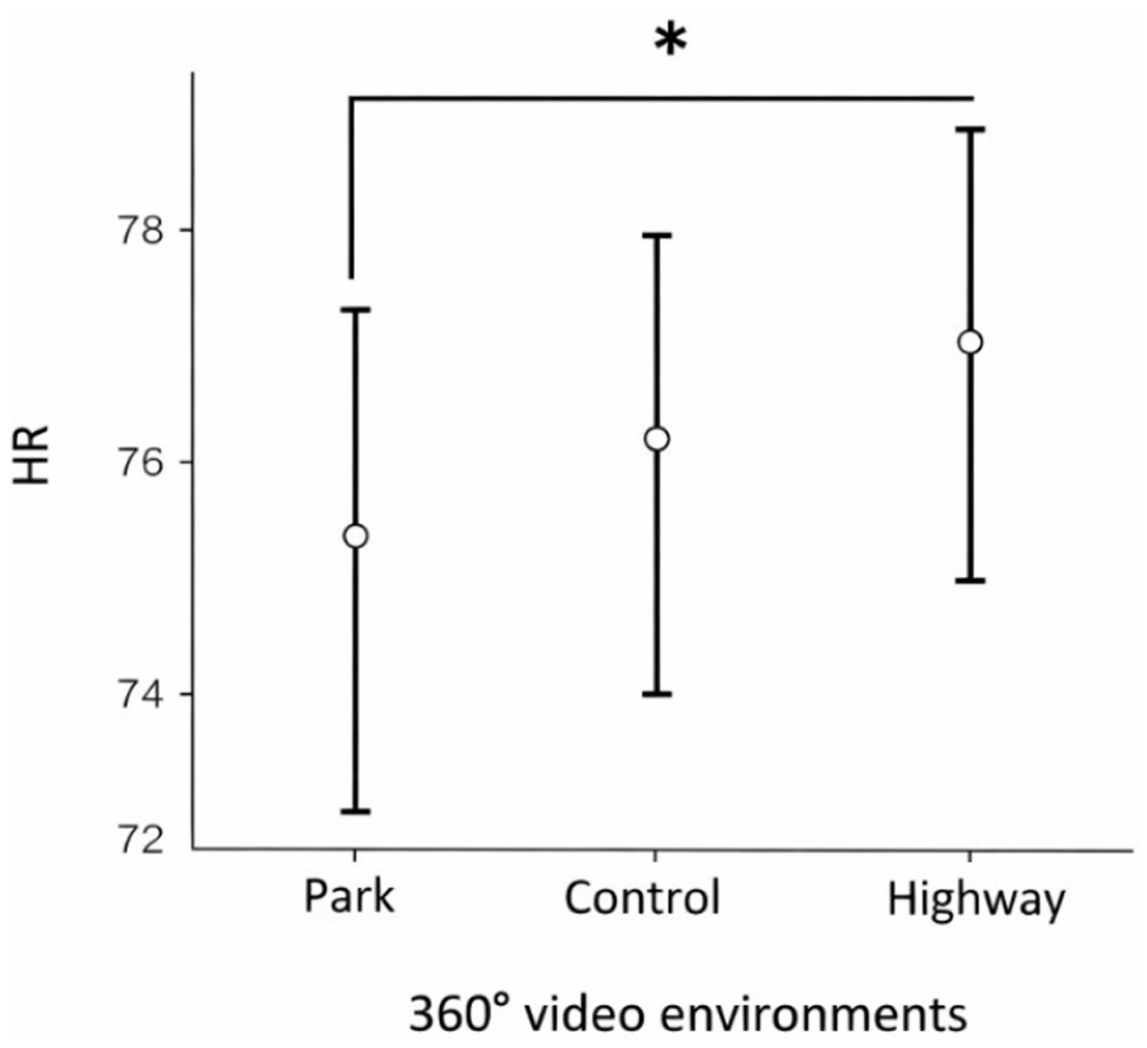
Estimated marginal mean heart rate (HR) recorded during exposure to 360° monoscopic videos of three environments (park, control, and highway), averaged over the 5 min video exposure period. Asterisks (*) indicate statistically significant differences between conditions. Error bars represent ±1 standard error of the mean (SEM).

A one-way repeated-measures ANOVA revealed a significant effect of the Video environment on HR: *F* (2, 130) = 3.58, *p* = 0.031, η^2^_p_ = 0.052 (see Figure 4). Post hoc tests indicated that the HR during the video walk through the park was significantly lower than the HR during the video walk through the highway: *t* (65) = −2.78, *p* = 0.019, *d* = 0.34. The HR did not significantly differ between the control condition and either the park (*t* (65) = −1.59, *p* = 0.259, *d* = 0.20) or the highway (*t* (65) = 1.04, *p* = 0.553, *d* = 0.13). The ANOVA revealed no significant effect of the video environment on HRV measures, including SDNN: *F* (2, 130) = 1.91, *p* = 0.152, η^2^_p_ = 0.029; LF/HF, *F* (2, 130) = 0.26, *p* = 0.774, η^2^_p_ = 0.001; and SD1/SD2, *F* (2, 130) = 1.86, *p* = 0.159, η^2^_p_ = 0.028.

To address potential post-stress baseline differences, we conducted a supplementary change-score analysis as a robustness check. Paired-samples *t*-tests comparing baseline-adjusted physiological responses produced the same pattern of results as the primary analyses (see Appendix A).

#### 3.2.2 Skin conductance response

**Figure 5:**
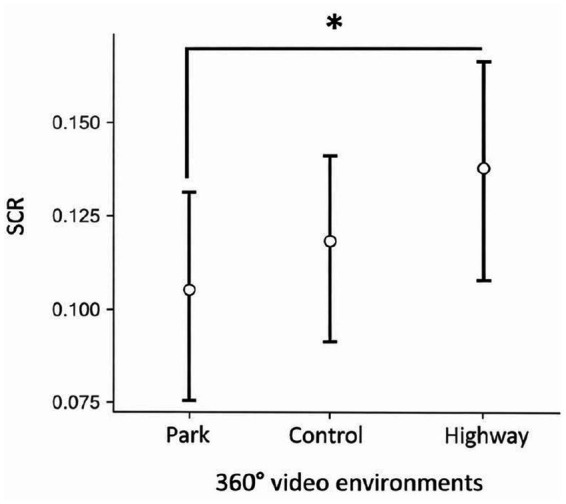
Estimated marginal means of skin conductance response (SCR) recorded during exposure to 360° monoscopic videos of three environments (park, control, and highway). Asterisks (*) indicate statistically significant differences between conditions. Error bars represent 1 standard error of the mean (SEM).

A one-way repeated-measures ANOVA revealed a significant effect of the Video environment on the SCR: *F* (2, 130) = 4.40, *p* = 0.014, η^2^_p_ = 0.063. *Post hoc* comparisons indicated that the SCR was significantly lower during the video walk through the park than during the video walk through the highway: *t* (65) = −2.65, *p* = 0.027, *d* = 0.33. Additionally, the SCR during the video walk through the highway did not significantly differ from SCR during exposure to the control video, *t* (65) = 0.41, *p* = 0.910, *d* = 0.05. No significant difference was found between the SCR during the video walk through the park and the control video (see Figure 5). Results were robust to baseline adjustment. Change-score analyses using paired-samples *t*-tests yielded the same pattern of findings (see Appendix A). Overall, these results support our hypothesis H1.

### 3.3 Risk-taking behavior (BART)

#### 3.3.1 Reward (total earnings)

A one-way repeated-measures ANOVA indicated no significant effect of stress induction on the total amount of money earned during the BART trials: *F* (2, 130) = 0.82, *p* = 0.369, η^2^_p_ = 0.012. Similarly, there was no significant effect of the Video environment on total earnings after stress: *F*(2, 130) = 0.92, *p* = 0.402, η^2^_p_ = 0.014. Post hoc tests confirmed that earnings did not significantly differ between the video walk through the park and the highway, *t* (65) = −1.30, *p* = 0.401, *d* = −0.16; the video walk through the park and the control condition, *t* (65) = −0.66, *p* = 0.788, *d* = 0.05; or the control condition and the video walk through the highway, *t* (65) = 0.72, *p* = 0.753, *d* = 0.09.

#### 3.3.2 Number of balloon pops

We found no significant difference between the number of balloon pops before (*M* = 31.2, *SD* = 9.12) and after (*M* = 30.7, *SD* = 8.74) stress induction during the BART trials. A one-way ANOVA indicated no significant effect: *F* (1, 65) = 2.27, *p* = 0.137, *η^2^p* = 0.034. The analysis of the Video environment factor, both before and after the BART trials, also revealed no significant effect on the number of balloon pops during the task: *F* (2, 130) = 0.09, *p* = 0.197, *η^2^p* = 0.001. Accordingly, *post hoc* tests showed no differences in the number of balloon pops between trials with video walks through the urban park and the highway: *t* (65) = 0.39, *p* = 0.921, *d* = 0.05; the video walks through the park and the control condition, *t* (65) = −0.38, *p* = 0.924, *d* = −0.05; or the video walks through the highway and the control condition, *t* (65) = −0.09, *p* = 0.996, *d* = −0.01.

#### 3.3.3 Number of balloon inflation pumps

Similarly, no significant differences were observed in the number of balloon inflation pumps before (*M* = 362, *SD* = 65.1) or after (*M* = 357, *SD* = 66.4) stress induction. A one-way repeated measures ANOVA confirmed that stress induction did not significantly impact the number of balloon inflation pumps: *F* (2, 130) = 2.60, *p* = 0.112, *η^2^p* = 0.038. The Video environment also did not have a significant effect on the number of balloon inflation pumps: *F* (2, 130) = 1.28, *p* = 0.282, *η^2^p* = 0.019. Post hoc tests showed no significant differences in the number of balloon inflation pumps between the Video park and highway walks, *t* (65) = −1.303, *p* = 0.399, *d* = −0.160, the VR park walk and the control condition, *t* (65) = 0.383, *p* = 0.923, *d* = 0.047, or the control condition and the Video highway walk: *t* (65) = −1.408, *p* = 0.343, *d* = 0.173. Therefore, these results disconfirm hypothesis H2.

## 4 Discussion

In this study, participants experienced a standardized physiological stressor induced by unpredictable electrical shocks and subsequently recovered in one of three 5-min 360° (monoscopic) video with VR headset: an urban walk through a green urban park, a built-up highway environment, or a minimal-stimulation control condition. Our results partially supported Hypothesis H1 and demonstrated that, relative to the built urban highway environment, exposure to the urban park facilitated more effective stress recovery. Specifically, participants exhibited significantly lower heart rate (HR) and skin conductance responses (SCR) during the park condition compared to the highway condition. In contrast, physiological responses during the park condition did not significantly differ from those observed during the control condition. Self-reported stress, however, was significantly lower following exposure to the park environment compared to both the highway and control conditions. Together, these findings suggest that immersive exposure to urban greenery may support subjective and physiological stress recovery, particularly when contrasted with highly arousing built urban environments.

In this study, we measured PPG to estimate HRV. However, PPG has been criticized as a less accurate method for HRV measurement compared to electrocardiograms (Heo et al., 2021), which may explain the lack of significant effects of the urban environment conditions on the participants’ HRV during stress recovery in our study. However, SCR, HR, and self-reports clearly indicated a more effective stress reduction, specifically with the 360° monoscopic videos of green urban environments. To our knowledge, this is the first study to combine 360° videos of urban walks in a real park and highway with the ToS paradigm to confirm the positive effects of urban green environments on stress recovery.

Our findings further confirm that green environments can reduce stress and positively affect daily life stress for city residents (Hedblom et al., 2019; Yin et al., 2020). Previous studies have suggested that stress-induced HR and SCR responses are more effectively reduced in green areas than in built urban areas (Hedblom et al., 2019; Hong et al., 2019; Van den Berg et al., 2015b; Jin et al., 2022). Importantly, our behavioral and psychophysiological findings add to the growing evidence that immersive videos of green areas, including nature, landscapes, and parks, are effective in reducing reported stress (Kim et al., 2021; Chen et al., 2025) and HR (Chan et al., 2023). Similar to previous studies (Valtchanov and Ellard, 2010; Hedblom et al., 2019; Huang et al., 2020), we found a stronger reduction of stress-induced SCR after exposure to 360° videos of the green area than after exposure to 360° videos of the built urban area. This further confirms that the immersive video park environment experience facilitates human stress recovery. Overall, we suggest that depicting real nature may have restorative characteristics, and immersing people in a virtual nature setting might alleviate stress. Although the 360° monoscopic video stimuli provided an immersive visual experience, presence and embodiment were not directly measured; therefore, conclusions regarding immersive engagement should be interpreted cautiously.

Our results also support the practical value of 360° video-based nature interventions. We found a substantial effect of 360° videos of parks on self-reported stress (the partial eta-squared = 0.302 and Cohen’s d (for the pairwise comparison between the highway and park conditions) = 1.01). Thus, 5-min exposures to immersive urban greenery can produce substantial psychological relief. Such findings highlight the powerful restorative potential of virtual nature and can be relevant for practical applications in clinical, educational, or workplace settings where access to natural green space is limited. Interaction with nature has been linked to decreased levels of stress, anxiety, depression, and perceived psychological distress (Haluza et al., 2014; Borgi et al., 2023; Tyrväinen et al., 2014). Spending time in real green environments enhances positive emotions, reduces negative emotions, and improves overall mood and well-being (Barton and Pretty, 2010; Lee et al., 2011). Our results further support the idea that virtual walks with spherical monoscopic video in green environments can be effectively used to relieve stress in uncomfortable urban environments that lack greenery. This underscores the potential benefits of investing in high-quality simulations for research on environmental experiences and stress reduction (Newman et al., 2022). Perhaps by making future 360° simulations of nature more interactive and multisensory, we could boost the effectiveness of video-based green interventions.

Unlike previous studies that predominantly focused on forests or lawns, the current study utilized 360° urban environment videos to create experiences that reflected life in real megacities. We also used a within-subject design to minimize the impact of individual differences among participants and included a control baseline condition with no visual stimulation. Importantly, the current study used the standard ToS paradigm, delivering infrequent and unpredictable electric shocks and evoking state anxiety. During such threat periods, participants reliably demonstrate increases in the subjective and physiological signatures of anxiety (e.g., Robinson et al., 2013). Anticipation of a future threat—anxiety—plays a unique role in the stress response. This type of anxiety-inducing stress is particularly relevant in urban environments, which constantly triggers expectations of negative outcomes. Our results are in line with studies showing that a higher percentage of green spaces lowers the chance of anxiety disorders (Maas et al., 2009; Kirubasankar et al., 2021; McKenzie et al., 2013). We also further support previous findings that immersive green environment interventions can significantly reduce the negative effects of anxiety (O’Meara et al., 2020).

Our results did not confirm hypothesis H2 and showed no influence of the 360° video of the urban park on risk-taking. Using BART, we found no significant difference in risk-taking behavior in the BART trial after video exposure to the park, highway, or control conditions. An extensive meta- analysis suggested that acute stress leads to riskier decisions (Starcke and Brand, 2012). However, the literature on the effects of acute stress on risk-taking is contradictory. Some studies have found an effect of stress on risk-taking only for males but not for females (Daughters et al., 2013; Lighthall et al., 2012). Another study reported increased risk-taking after stress only for participants who suffered social anxiety but not for healthy individuals (Reynolds et al., 2013). Interestingly, Lighthall et al. (2012) reported no stress-related differences in risk-taking, while Finy et al. (2014) reported decreased risk-taking under stress for both genders. A recent study showed that stress induction increased participants’ levels of cortisol, arousal, and systolic blood pressure but did not affect risky decision-making (Von Helversen and Rieskamp, 2020). Furthermore, a moderation analysis suggested that the effect of stress on risk-taking depends on the interaction of affective response and cortisol reactivity. Thus, future studies should collect more data on the physiological and affective markers of stress responses to clarify the interaction between stress and urban environments during risk-taking. Researchers should also employ other risk-taking paradigms to study the effect of the urban environment on risk-taking in different domains, including health behavior.

### 4.1 Limitations

We used monoscopic panoramic videos of actual urban environments presented via a head-mounted display. Despite the advantages of this naturalistic approach, future studies should more precisely manipulate and control stimulus characteristics such as visual complexity and overall information load. In the present study, the black-screen control condition differed from the park and highway videos not only in environmental content but also in visual stimulation. Therefore, reduced physiological responses in the control condition may partly reflect low cognitive load or boredom rather than restorative processes per se. This distinction limits the interpretation of park–control comparisons. Unlike complex urban environments, our control condition may induce sensory deprivation. Thus, future studies should use more ecologically valid control stimuli, such as real-world neutral (urban) videos, to better isolate the restorative effects of urban greenery.

To control the soundscape, all videos were presented without auditory input. The exclusion of sound could create a sensory mismatch, particularly in highway conditions where active traffic is not paired with a prominent traffic notice. Such unnatural stimuli might induce an additional cognitive load, affect stress reduction, and lower ecological validity. Thus, our study design may influence stress recovery dynamics and reduce ecological validity, particularly for urban environments such as highways, where sound is a salient real-world stressor. Future studies should incorporate well-controlled multisensory cues to more fully capture real-world environmental experiences. Additionally, to minimize VR sickness in our study, participants viewed short (5-min) smooth-motion videos while seated (for a similar approach, see Yin et al., 2020). Nonetheless, future research should clarify how simulator sickness may affect longer immersive nature interventions.

We used the Balloon Analogue Risk Task (BART) to assess risk-taking behavior. Prior research suggests that associations between impulsivity and laboratory-based risk tasks such as BART are modest (Elliott et al., 2023); thus, future studies may benefit from incorporating additional behavioral tasks or real-world measures of risk-taking. Finally, heart rate (HR) and heart rate variability (HRV) were measured using photoplethysmography (PPG). Although PPG is a well-established, non-invasive method well-suited for VR settings, it is less precise than electrocardiography (ECG), which may have contributed to the null findings for HRV. Future studies of green interventions could incorporate ECG or more advanced biosensors. Nevertheless, these limitations do not undermine our key findings that exposure to the 360° monoscopic urban park videos led to lower HR, reduced SCR, and lower self-reported stress compared to the highway or control conditions.

## 5 Conclusion

This study explored whether brief, immersive 360° videos of urban walks could support stress recovery following acute stress. Using a threat-of-shock paradigm, we found that participants reported significantly lower stress after viewing a virtual walk through an urban park compared to both the highway and control conditions. At the physiological level, heart rate and skin conductance responses were lower during exposure to the immersive park environment than during exposure to the highway environment, whereas no significant physiological differences were observed between the park and control conditions. Heart rate variability and risk-taking behavior, assessed using the Balloon Analogue Risk Task, were not affected by the virtual environments.

Taken together, these findings suggest that short exposures to virtual urban green spaces can support stress recovery, particularly when contrasted with highly stimulating urban environments. The effects were more robust for subjective stress than for physiological measures, highlighting potential differences in sensitivity across outcome domains. It is also important to note that the control condition consisted of a minimal-stimulation (non-video) display rather than a neutral urban video, which may have reduced sensory engagement and contributed to the absence of physiological differences between the park and control conditions. Future research should employ more ecologically matched control stimuli better to better isolate the restorative effects of immersive green environments and to clarify how different levels of sensory stimulation influence stress recovery in urban contexts.

## Data Availability

The original contributions presented in the study are included in the article/supplementary material, further inquiries can be directed to the corresponding author.

## Appendix A

### Baseline-adjusted change score analyses

Please find the 360-degree monoscopic videos at the following link: VR Videos.

To evaluate whether observed physiological differences between environmental conditions were influenced by post-stress baseline variation, we conducted supplementary analyses using change scores rather than absolute physiological values. The baseline period consisted of a neutral black-screen display presented immediately following the stress induction, providing a low-stimulation reference condition for physiological normalization.

For each participant, change scores were calculated by subtracting physiological values recorded during the black-screen baseline from values recorded during the VR exposure. Paired-samples t-tests were used to compare baseline-adjusted physiological responses between the park and Highway conditions.

Results indicated significantly greater physiological restoration in the park relative to the Highway condition for heart rate, t (65) = 2.78, *p* = 0.007, Cohen’s d = 0.34, and skin conductance, t (65) = −2.65, *p* = 0.010, Cohen’s d = −0.33. In contrast, baseline-adjusted differences for the LF/HF ratio, t (65) = −0.79, *p* = 0.433, and SD12, t (65) = 0.14, *p* = 0.891, were not statistically significant.

Overall, these baseline-adjusted analyses corroborate the primary results reported in the main text and indicate that the observed restorative effects of the park environment on heart rate and skin conductance are robust to post-stress baseline variation.
